# Supplementary material for: Kidney Injury Caused by Preeclamptic Pregnancy Recovers Postpartum in a Transgenic Rat Model
Source: Int J Mol Sci. 2021 Apr 5;22(7):3762. doi: 10.3390/ijms22073762 (PMC8038582; doi:10.3390/ijms22073762)
Supplement: Supplementary file 1 [file ijms-22-03762-s001.pdf]

Supplementary Material

Table S1. Primer pairs and probes used for RT-qPCR.

Nucleotide sequences are indicated in 5' to 3' direction. All Primers/probes were ordered from BioTeZ Berlin Buch GmbH. A = deoxy adenosine triphosphate; C = deoxy cytosine triphosphate; G = deoxy guanine triphosphate; T = deoxy thymine triphosphate.

**Gene Abbr.**	**Name**	**Sequence forward primer**<br>**Sequence reverse primer**<br>**Sequence probe (all linked to 5' Fam and 3' Tamra)**
18S	18s	5´ ACA TCC AAG GAA GGC AGC AG 3´
		5´ TTT TCG TCA CTA CCT CCC CG 3´
		5´ CGCGCAAATTACCCACTCCCGAC 3´
CD68	Cd68 molecule	5' CACTTCGGGCCATGCTTCT 3'
		5' GGAGGACCAGGCCAATGAT 3'
Col-1	collagen type 1 alpha 1 chain	5' AGA GCG GAG AGT ACT GGA 3'
		5' CTG ACC TGT CTC CAT GTT GCA 3'
		5´ CAA GGC TGC AAC CTG GAT GCC ATC 3´
Col4α-1	collagen type 4 alpha 1 chain	5´ ATC CGG CCC TTC ATT AGC A 3'
		5´ GAC TGT GCA CCG CCA TCA C 3'
		5´ TTT GCG AAG CGC CCG CCA 3´
Ctgf	connective tissue	5´ CGC CAA CCG CAA GAT TG 3´
		5´ CAC GGA CCC ACC GAA GAC 3´

	growth factor	5´ CAC TGC CAA AGA TGG TGC ACC CTG 3´
Cyb-α	cytochrome b-245, alpha chain polypeptide	5´ CTC TAT TGT TGC AGG AGT GCT CAT 3´
		5´ GGT GGA GCC CTT TTT CCT CTT 3´
		5´ TGT CTG CTG GAG TAC CCC CGG G 3´
Cyb-β	cytochrome b-245, beta chain	5´ GGA GTG CCC AGT ACC AAA GTT T 3´
		5´ GAA CAT GGG ACC CAC TAT CCA 3´
		5´ CCG GAA ACC CTC CTA TGA CTT GG 3´
Et-1	endothelin 1	5' AGG GAA AAC CCT GTC CCA AG 3'
		5' CAC GGG GCT CTG TAG TCA AT 3'
Flk-1 (VEGFR-2)	kinase insert domain receptor	5´ ACA GCA TCA CCA GCA GTC AG 3'
		5´ CCA AGA ACT CCA TGC CCT TA 3'
Flt-1 (VEGFR-1)	FMS-related tyrosine kinase 1 (VEGF receptor)	5´ TCC CTC AGC CTA CCA TCA AG 3'
		5´ GAG AGT CAG CCA CCA CCA AT 3'
Kim-1	kidney injury molecule 1	5' ATA ATC ACA CTG TAA GAA TCC CTT TGA G 3'
		5' CAA CGG ACA TGC CAA CAT AGA 3'
		5' CCG CGA AGA AAC CCG ACT AAG GGC 3´

Mcp-1	monocyte chemoattractant protein 1	5' TGC AGT TAA TGC CCC ACT CA 3'
		5' TCT CCA GCC GAC TCA TTG G 3'
Ncf-1	neutrophil cytosolic factor 1	5´ AGA GAC ATA CCT GAC GGC CAA A 3´
		5´ GGC CCG ATA GGT CTG AAG GA 3´
		5´ ATG GCA AGA ATA ATG TAG CTG ACA TCA CGG G 3`
Ncf-4	neutrophil cytosolic factor 4	5´ TGG GTG TCA AAC AAG AGA TTG C 3´
		5´ GGC TCA GGA GGT TCT TCA TGT AG 3´
		5´ AGA CTC GGA TCC CGG CCC TCA A 3´
Ngal	neutrophil gelatinase associated lipocalin 2	5´ TCG TCA GCT TTG CCA AGT C 3'
		5´ TTG GTC GGT GGG AAC AGA 3'
		5´ TGG GCC TCA AGG ATA ACA ACA TCG TTT 3´
Nox-4	NADPH oxidase 4	5´ GCC TGA TCC TTT TAC CCA TGT G 3´
		5´ CCT GCT AGG GAC CTT CTG TGA 3´
		5´ CACAGTCCTGGCTTACCTTCGCGG 3´

Nphs-1	nephrin	5´ CAA AAA TGT ATC ACA CCA AAG GAC AA 3´
		5´ AAC ACA ATC CTG AGG CAC AGT CT 3´
		5´ AAG GTT CTG TTT GTC TCC GGT CA 3´
sFlt-1	soluble FMS-related tyrosine kinase-1	5´ GGG AAG AGA TCC TTC GGA AGA 3´
		5´ GAG ATC CGA GAG AAA ATA GCC TTT T 3´
		5´ AGA AGT TCT CGT TAG AGG TGA GCA CTG CAG C 3´
Tgf-β*1*	transforming growth factor beta 1	5' GCC TGA GTG GCT GTC TTT TGA 3'
		5' CCT GTA TTC CGT CTC CTT GGT T 3'
		5' CAC TGG AGT TGT CCG GCA GTG GC 3'
Vegf-a	Vascular endothelial growth factor a	5´ GGG CTG CTG CAA TGA TGA A 3'
		5´ TCC GCA TGA TCT GCA TAG TGA 3'
Vegf-c	Vascular endothelial growth factor c	5´ TCA GCA AGA CGT TGT TTG AAA TT A 3'
		5´ CAG GAA TGA TTG GCA AAA CT 3'

Table S2. Relative gene expression of marker genes.

Gene expression is normalized to 18s expression. Markers showed no significant differences in two-way ANOVA. Sidak's multiple comparison was performed Mean (n = 5-7) ± SD is shown. Adjusted p-value is indicated. d = day. WT = wild type, PE = preeclampsia.

**Gene**	**Time Point**	**mean relative expression WT ± SD**	**mean relative expression PE ± SD**	**P-value (adjusted)**
Kim-1	d21	0.08 ± 0.021	4.14 ± 3.262	< 0.001
	d50	0.23 ± 0.125	0.09 ± 0.050	0.999
Ngal	d21	0.59 ± 0.279	1.64 ± 0.825	< 0.001
	d50	0.47 ± 0.108	0.37 ± 0.172	0.880
Nphs-1	d21	0.89 ± 0.228	0.97 ± 0.273	0.932
	d50	0.67 ± 0.228	0.65 ± 0.091	> 0.999
Et-1	d21	0.77 ± 0.159	0.83 ± 0.143	0.830
	d50	0.54 ± 0.130	0.68 ± 0.259	0.338
Flt-1	d21	1.03 ± 0.117	1.13 ± 0.137	0.402
	d50	0.85 ± 0.151	0.85 ± 0.147	0.999
Flk-1	d21	0.87 ± 0.167	1.03 ± 0.091	0.069
	d50	0.68 ± 0.105	0.81 ± 0.108	0.128
sFlt-1	d21	0.89 ± 0.179	0.85 ± 0.136	0.908
	d50	0.69 ± 0.139	0.59 ± 0.154	0.410
Vegf-a	d21	0.86 ± 0.151	0.96 ± 0.101	0.442

	d50	0.77 ± 0.088	0.87 ± 0.175	0.301
Vegf-c	d21	0.71 ± 0.159	0.65 ± 0.143	0.726
	d50	0.59 ± 0.084	0.74 ± 0.113	0.059
Col-1	d21	1.03 ± 0.418	0.67 ± 0.158	0.043
	d50	0.67 ± 0.107	0.57 ± 0.146	0.675
Col4α-1	d21	0.79 ± 0.197	0.90 ± 0.119	0.253
	d50	0.63 ± 0.078	0.61 ± 0.082	0.946
Ctgf	d21	0.92 ± 0.318	1.51 ± 0.462	0.011
	d50	0.80 ± 0.251	0.78 ± 0.246	0.986
Tgf-β1	d21	0.80 ± 0.188	0.99 ± 0.119	0.128
	d50	0.78 ± 0.144	0.76 ± 0.195	0.950
CD-68	d21	0.72 ± 0.176	1.02 ± 0.118	0.030
	d50	0.55 ± 0.14	0.81 ± 0.118	0.029
Mcp-1	d21	0.69 ± 0.463	0.94 ± 0.276	0.432
	d50	0.70 ± 0.272	0.58 ± 0.356	0.760
Cyb-a	d21	0.94 ± 0.099	0.94 ± 0.068	> 0.999
	d50	0.92 ± 0.090	0.95 ± 0.168	0.867
Cyb-b	d21	1.19 ± 0.322	1.31 ± 0.149	0.772
	d50	0.74 ± 0.140	0.98 ± 0.335	0.191
Ncf-1	d21	1.11 ± 0.564	0.73 ± 0.080	0.094
	d50	0.61 ± 0.070	0.74 ± 0.198	0.687
Ncf-4	d21	0.77 ± 0.187	1.05 ± 0.157	0.011
	d50	0.63 ± 0.077	0.84 ± 0.157	0.024

Nox-4	d21	0.71 ± 0.125	0.62 ± 0.056	0.265
	d50	0.59 ± 0.058	0.84 ± 0.129	< 0.001

Figure S1:

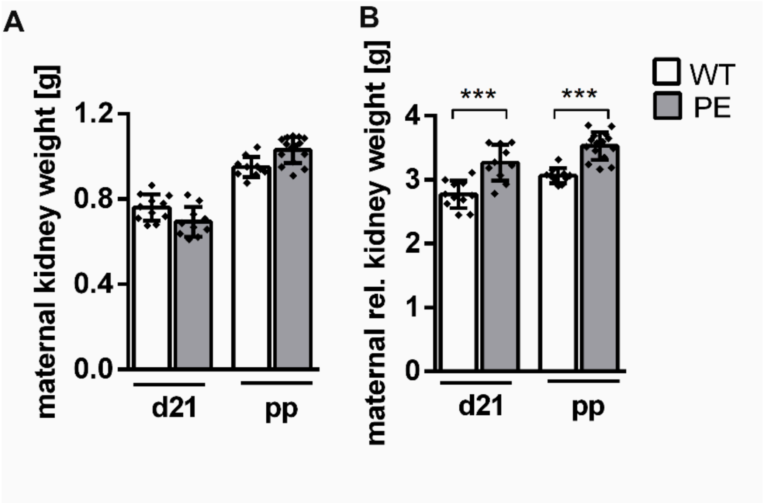

Maternal kidney weight in pregnancy and postpartum.

(A) Displayed is the kidney weight in grams (g) of Wildtype (WT) and preeclampsia (PE) rats during pregnancy (day 21) and postpartum pregnancy (d50). (B) The kidney weight was normalized to maternal body weight in grams (g). Statistical analysis was done with a two-way ANOVA. P-value is shown *** ≤ 0.001. Mean ± SD is indicated.
